# Extracellular adenosine triphosphate is associated with airflow limitation severity and symptoms burden in patients with chronic obstructive pulmonary disease

**DOI:** 10.1038/s41598-019-51855-w

**Published:** 2019-10-25

**Authors:** Iva Hlapčić, Andrea Hulina-Tomašković, Anita Somborac-Bačura, Marija Grdić Rajković, Andrea Vukić Dugac, Sanja Popović-Grle, Lada Rumora

**Affiliations:** 1 University of Zagreb, Faculty of Pharmacy and Biochemistry, Department of Medical Biochemistry and Haematology, Zagreb, Croatia; 2 University Hospital Centre Zagreb, Clinical Department for Lung Diseases Jordanovac, Zagreb, Croatia; 3 University of Zagreb, School of Medicine, Zagreb, Croatia

**Keywords:** Biochemistry, Cell biology, Inflammation

## Abstract

Extracellular adenosine triphosphate (eATP)-driven inflammation was observed in chronic obstructive pulmonary disease (COPD) but was not investigated in patients’ blood. Therefore, this study aimed to investigate eATP concentration in plasma of COPD patients and its association with disease severity and smoking. Study included 137 patients with stable COPD and 95 control subjects. eATP concentration was determined in EDTA plasma by luminometric method, and mRNA expression of eATP receptors P2X7R and P2Y2R was analysed by quantitative polymerase chain reaction (qPCR). eATP concentration was increased in COPD patients compared to controls (P < 0.001). Moreover, it was increasing with disease severity (GOLD 2–4) as well as symptoms burden and exacerbations history (GOLD A–D) (P < 0.05). eATP in healthy smokers differed from healthy non-smokers (P < 0.05) but was similar to GOLD 2 and GOLD A patients. eATP showed great diagnostic performances (OR = 12.98, P < 0.001) and correctly classified 79% of study participants. It demonstrated association with FEV_1_ and multicomponent indices (ADO, BODEx, BODCAT, CODEx, DOSE). Regarding gene expression, P2Y2R was increased in the blood of COPD patients. Plasma eATP could become a diagnostic and/or prognostic biomarker in COPD, as it seems to be associated with patients’ condition, quality of life and disease progression.

## Introduction

Adenosine triphosphate (ATP) might be an important molecule in the pathogenesis of airway diseases due to its secretion by activated macrophages, neutrophils, epithelial cells, endothelial cells and platelets^1,2^. Once released in extracellular space, ATP contributes to the exacerbation of inflammation, bronchoconstriction and cough. Moreover, it modulates airway hypersensitivity and immune cells function^3^. Therefore, if there is an accumulation of extracellular ATP (eATP) in the airways, patients are prone to pro-inflammatory responses^4^. ATP is present in extracellular matrix in lower concentrations when inflammation is induced as a part of normal physiological response. However, eATP can be found in higher concentrations when cell death or cell activation are stimulated^2,5,6^.

Most airway diseases are associated with ATP accumulation in bronchoalveolar lavage (BAL), sputum or exhaled breath condensate (EBC). In general, BAL samples reflect only the peripheral airways, sputum represents situation in the central airways, while the origins and the mechanism of airway secretions in EBC are not fully understood^7^. Chronic obstructive respiratory disease (COPD) is a heterogeneous and complex disease characterized by airway obstruction and inflammation^8^. Increased eATP concentrations were determined in the airways of patients with COPD when measured in BAL, and the highest concentration was observed in COPD patients with more advanced disease^9^.

Although inflammation in lungs can be caused by different harmful particles, cigarette smoke is considered the most common risk factor for development of COPD^10^. The effect of cigarette smoke on ATP release was investigated in a mouse model of smoke-induced acute lung inflammation and emphysema, and an increase in eATP was found in their BAL^11^. The effect of smoking was investigated in human samples, too. Neutrophils were isolated from blood of healthy non-smoking donors and stimulated with cigarette smoke. Afterwards, concentration of eATP was measured and it was increased in comparison to non-stimulated neutrophils^12^. It was also shown that higher eATP concentrations were present in BAL of COPD patients compared to healthy subjects, even after smoking cessation^9,13^. However, when ATP was measured in EBC, no differences between healthy non-smokers, healthy smokers and COPD patients were found^7^. Nevertheless, to the best of our knowledge, eATP concentrations in COPD patients’ blood were not determined so far.

ATP is a ligand for some of the purinergic receptors (P2Rs) that are widely expressed in the lungs – trans-cell membrane cationic channels (P2XRs) and trans-membrane domain G protein-coupled receptors (P2YRs). When ATP binds to the P2Rs, macrophages and neutrophils secrete pro-inflammatory molecules and mediators of tissue degradation, all of which contribute to the chronic inflammation in COPD^13^. There are seven P2XRs (P2X_1–7_R) and eight P2YRs (P2Y_1/2/4/6/11/12/13/14_R). Common receptors investigated from those two families are P2X7R and P2Y2R^14,15^. P2X7R receptors are predominantly intracellular and they locate to the plasma membrane upon differentiation of monocytes to macrophages^16^. Once bound to P2X7R, eATP induces NLRP3 inflammasome activation that leads to the maturation and release of IL-1β^17^. Macrophages from BAL and blood neutrophils of COPD patients showed higher expression of the P2X7R^11^. While P2X7R is activated only by ATP, P2Y2R can be activated by ATP and UTP^3,18^. Nevertheless, it is considered that activated P2Y2Rs have a role in directing neutrophil chemotaxis and amplifying chemotaxis signals, so eATP can be an autocrine and paracrine messenger^5,15,19^.

This study aims to investigate for the first time eATP in peripheral blood of COPD patients. Pattern of eATP fluctuation associated with smoking, severity of airflow limitation (Global Initiative for Chronic Obstructive Lung Disease (GOLD) 1–4 classification assessment) as well as symptoms and history of exacerbations (GOLD A–D classification assessment) was also determined, as none of this was explored so far. Finally, mRNA expression level of P2X7R and P2Y2R, two common eATP receptors that could have a role in COPD pathogenesis associated with eATP, was investigated. We hypothesized that eATP would be elevated in peripheral blood of COPD patients when compared to age- and sex-matched control subjects, and related to smoking status as well as severity of disease.

## Methods

### Participants of the study

There were 232 participants in this retrospective study. 95 were in control group and 137 were COPD patients in stable phase of the disease. They were recruited at the Clinical Department for Lung Diseases Jordanovac, University Hospital Centre Zagreb (Zagreb, Croatia), signed an informed consent for scientific research and agreed to volunteer. Ethical Committee of University Hospital Centre Zagreb and Ethical Committee for Experimentation of Faculty of Pharmacy and Biochemistry, University of Zagreb (Zagreb, Croatia), approved the study (Approval Protocol Numbers: 02/21/JG and 251-62-03-14-78, respectively).

COPD was confirmed by pulmonologists according to the ratio of spirometry parameters forced expiratory volume in one second (FEV_1_) and forced vital capacity (FVC) measured in litres (FEV_1_/FVC <0.70), as described by the GOLD guidelines^8^. Stable phase of the disease was defined as no exacerbations in the last three months, no changes in medications for respiratory system and no symptoms of infection in lower respiratory tract. Exclusion criteria for COPD patients were as follows: age under 40, lung diseases other than COPD, inflammatory systemic diseases, acute infections, diabetes with severe complications, severe liver diseases, severe kidney insufficiency, malignant diseases, transplantations, and other specific or non-specific acute inflammations.

Health state of control subjects was established based on anamnestic data and spirometry test results. Control individuals had to meet the same inclusion and exclusion criteria as COPD patients, except for the findings of post-bronchodilator spirometry test results (that were normal for control subjects). They were age- and sex-matched to their COPD counterparts, living in the same area of Croatia as COPD patients.

Both control and COPD groups of patients were subdivided according to the smoking status, so there were non-smokers (n = 48) and smokers (n = 47) in control group, and non-smokers (n = 10), former smokers (n = 90) and smokers (n = 37) in COPD group of patients. Moreover, COPD patients were classified into different GOLD stages based on FEV_1_, as recommended by GOLD: GOLD 1 (FEV_1_ ≥ 80%) (n = 0), GOLD 2 (50% ≤ FEV_1_ < 80%) (n = 47), GOLD 3 (30% ≤ FEV_1_ < 50%) (n = 50) and GOLD 4 (FEV_1_ < 30%) (n = 40) stage of the disease. Nevertheless, besides airflow limitation severity, COPD patients were distinguished in the groups according to the symptoms and history of exacerbations (ABCD assessment): GOLD A (n = 27), GOLD B (n = 70), GOLD C (n = 0) and GOLD D (n = 40). COPD patients completed both Modified Medical Research Council (mMRC) Dyspnoea Scale and COPD Assessment Test (CAT) questionnaires. Body mass index (BMI), number of exacerbations during previous year and Charlson comorbidity index were additionally matched to every COPD patient. Afterwards, some multicomponent indices already established for evaluation of patient’s condition were calculated: ADO, BODCAT, BODEx, CODEx and DOSE^20–23^. ADO is composed of age, dyspnoea, and airflow obstruction; BODCAT is composed of BMI, airflow obstruction, dyspnoea, and CAT score; BODEx is composed of BMI, airflow obstruction, dyspnoea, and previous exacerbations; CODEx is composed of comorbidities (Charlson index), airflow obstruction, dyspnoea, and previous exacerbations; DOSE is composed of dyspnoea, airflow obstruction, smoking status, and previous exacerbations. Previous exacerbations were defined as a number of exacerbations in the previous year.

### Spirometry

Spirometry is a common method in diagnosing the airflow limitation. The spirometry was performed in outpatient clinic by trained technicians on each visit for COPD patients and once for control subjects. The spirometry was done on a MasterLab (Jaeger, Würzburg, Germany), according to the recommendations of the European Respiratory Society and American Thoracic Society. Spirometry was repeated at least three times, sometimes eight times, until two reproducible efforts were obtained. The exhalation effort had to be at least 6 seconds, or until the end-expiratory plateau was reached. The two largest FVC and FEV_1_ values had to show less than 5% variability, according to the standardized procedure^24^. Predicted values were the most commonly used European Community of Coal and Steel (ECCS) values^25^. The lung function parameters measured were FEV_1_, FVC and FEV_1_/FVC. A bronchodilator test with 400 µg of salbutamol was taken for each individual. Spirometry measurements were performed 15–30 minutes after salbutamol application. The same rules and standards were applied in prebronhodilator spirometry, and the best values for FEV_1_ and FVC were chosen.

### Diffusion capacity for carbon monoxide (DLCO) measurement

Pulmonary diffusing capacity was measured by single-breath method with carbon monoxide, on MasterScreen PFT Pro (Jaeger, Würzburg, Germany), according to the guidelines^26^. Helium dilution was used to determine alveolar volume (VA) and calculate the transfer coefficient for carbon monoxide (KCO) - DLCO/VA, as a unit diffusion indicator. Measurement results were correlated with the subjects’ haemoglobin values. Each subject made three measurements. Predictive values were estimated according to Cotes^27^.

### Blood sampling and preparation

Blood samples were collected from 7 a.m. to 9 a.m. after overnight fasting by venepuncture of a large antecubital vein into two tubes (6 mL) with K_3_-ethylenediaminetetraacetic acid (K_3_EDTA) anticoagulant (Greiner Bio-One, GmbH, Kremsmünster, Austria), and were mixed by an inversion for 8 times. For venepuncture, order of blood sampling and mixing, the guidelines were followed according to the national recommendations for venous blood sampling^28^. Samples were centrifuged immediately after blood collection for the two times as follows - firstly, 10 minutes at 3500 rpm at +4 °C and secondly, 15 minutes at 4000 rpm at +4 °C, as recommended by the Clinical and Laboratory Standards Institute (CLSI) guidelines^29^. After first centrifugation, buffy coat layers were removed into two 2 mL tubes with 1 mL of TRI Reagent Solution (Thermo Fischer Scientific, Waltham, Massachusetts, USA) and stored at −80 °C for isolation of RNA. After second centrifugation, remaining EDTA plasma was prepared for eATP determination. Those EDTA plasma samples were immediately mixed (2:1) with ATPlite Mammalian Cell Lysis Solution from the ATPlite kit for measurement of ATP (Perkin Elmer, Waltham, Massachusetts, USA), briefly mixed on vortex, and stored at −80 °C until analysis. ATPlite Mammalian Cell Lysis Solution was added to inactivate endogenous ATPases and to stabilize eATP, as suggested by manufacturer.

### Relative mRNA expression

Total RNA was isolated from buffy coats stored with TRI Reagent Solution by conventional method^30^. Concentration of RNA was measured by Nanodrop 8000 (Thermo Fischer Scientific, Wilmington, USA) and its quality was assessed by A_260_/A_280_ ratio with criteria A_260_/A_280_ = 1.9–2.1. cDNA was prepared with random primers and RevertAid First Strand cDNA Synthesis Kit (Thermo Fischer Scientific, Waltham, Massachusetts, USA) by GeneAmp PCR System 9700 (Applied Biosystems, Foster City, USA), with PCR conditions being 5 min at 25 °C, 60 min at 42 °C and 5 min at 70 °C. Quantitative polymerase chain reaction (qPCR) was performed with Taqman Universal PCR Mastermix (Applied Biosystems, Foster City, USA), and unlabelled PCR primers and Taqman probe from Taqman Gene Expression Assays (Applied Biosystems, Foster City, USA). PCR conditions were 2 min at 50 °C, 10 min at 95 °C, followed by 40 cycles of 15 seconds at 95 °C and 60 °C for 1 minute, using the 7500 Real-Time PCR System (Applied Biosystems, Foster City, USA). For the detection of mRNA expression, specific primers to P2X7R (Hs00175721_m1; Applied Biosystems, Foster City, USA) and P2Y2R (Hs04176264_s1; Applied Biosystems, Foster City, USA) were used. Beta-2-microglobulin (B2M) (Hs99999907_m1; Applied Biosystems, Foster City, USA) and peptidylprolyl isomerase (PPIA) (Hs99999904_m1; Applied Biosystems, Foster City, USA) were used as reference genes and were performed during each run for each sample for the normalization between the samples. Results of gene expression in controls and COPD patients were compared to the results of a randomly selected healthy control’s sample that was included in every plate as internal control. Data were expressed as a fold change of mRNA after following calculation ∆∆Ct = mean ∆Ct value (target samples) – mean ∆Ct value (control samples), where fold change value corresponds to the 2^−∆∆Ct ^^31^.

### Measurement of ATP

ATP levels were measured using ATPlite assay (Perkin Elmer, Waltham, Massachusetts, USA), according to the instructions of the manufacturer (ATPlite Luminescence ATP Detection Assay System, Rev. F – April 2015), with some modifications due to the human plasma used as a sample.

Dilutions of ATP standards (0.016–2 μM) and prepared plasma samples (1:10) were done with water. The luminescence in white 96-well plates was measured by the multilabel plate reader Victor 3 (Perkin Elmer, Waltham, Massachusetts, USA). Afterwards, four parameter logistic curve fit was used for calculating ATP concentrations by OriginPro 9 software program (OriginLab Corporation, Northampton, Massachusetts, USA). We determined intra-assay and inter-assay variations for the measurement of ATP in human EDTA plasma. Intra-assay variation analysis was run with a randomly selected healthy control’s sample at one plate, and coefficient of variation (CV) for plasma ATP concentration was 7.23% (n = 20). Inter-assay variation analysis was performed with the same randomly chosen healthy control’s sample, which was included as internal control at every plate run with participants’ samples. Inter-assay CV was 10.38% (n = 14).

### Statistics

Data were tested for normality by Kolmogorov-Smirnov test. As all data failed a normality test, a nonparametric Mann-Whitney test was used for the analysis of the difference between controls and COPD. Kruskal-Wallis test followed by a post-hoc analysis was used in a case of comparison of more than two groups. Chi-squared test was used for comparison of categorical variable (sex). For determination of association between the parameters, Spearman Rank Order was performed^32^. Univariate logistic regression analysis was also performed, and odds ratio (OR) with 95% confidence interval (95% CI) values were obtained. Results were considered statistically significant if P < 0.05. MedCalc statistical software version 17. 9. 2. (MedCalc Software, Ostend, Belgium) was used in the study.

## Results

Basic characteristics and lung function parameters of participants are shown in Table 1. There was no difference in age (P = 0.073) or sex (P = 0.118) between controls and COPD patients. However, spirometric variables were significantly lower in patients with COPD compared to healthy individuals, as expected (P < 0.001).

**Table 1:** Basic characteristics and lung function parameters of participants included in the study.

parameter	controls n = 95	COPD patients n = 137	P-value
age/years	64 (46–83)	65 (44–86)	0.073
**sex**			
males	49	86	0.118
females	46	51	
FEV_1_*/L	2.60 (2.12–3.19)	1.08 (0.78–1.57)	<0.001
FEV_1_/%	93.30 (86.38–104.20)	39.00 (28.08–59.73)	<0.001
FVC^▫^/L	3.35 (2.77–4.16)	2.28 (1.81–2.77)	<0.001
FEV_1_/FVC	0.81 (0.77–0.88)	0.48 (0.41–0.58)	<0.001

Age is presented as median with minimum – maximum, sex is presented as absolute number, while all other parameters are presented as median with interquartile range. Chi-squared test was used for comparison of males and females, while all other parameters were tested by Mann-Whitney test. Data were considered significant if P < 0.05.

*FEV_1_ – forced expiratory volume in 1 second; ^▫^FVC – forced vital capacity.

### Influence of smoking history on eATP concentration

**Figure 1:**
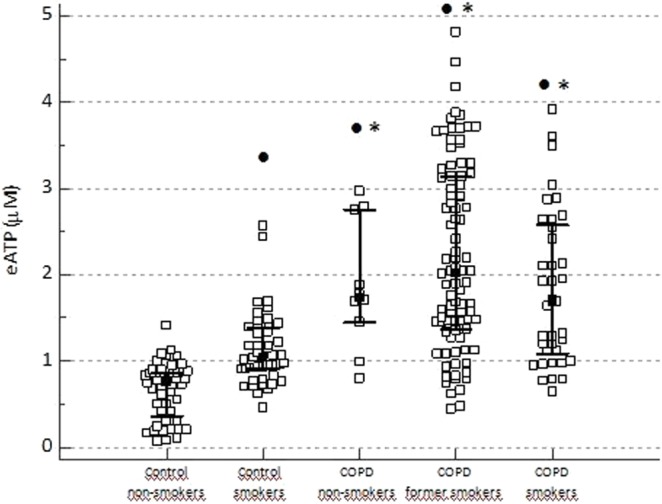
Influence of smoking history on eATP concentration determined in EDTA plasma of control non-smokers, control smokers, COPD non-smokers, COPD former smokers and COPD smokers. Data are shown as median with interquartile range for all the groups. Kruskal-Wallis test showed there was a significant difference between the groups (P < 0.001), and post-hoc analysis was performed. No significant difference was found between COPD patients subdivided according to their smoking status. ^•^Statistically significant difference in comparison to control non-smokers; *statistically significant difference in comparison to control smokers.

We found that eATP was significantly increased in plasma of patients with COPD compared to control subjects [1.879 (1.262–2.888) µM *vs*. 0.875 (0.701–1.074) µM, respectively; P < 0.001]. When participants were subdivided according to their smoking history, eATP concentration was found to be elevated in healthy smokers in comparison to healthy non-smokers (P < 0.05). Moreover, eATP was increased in total cohort of COPD patients in comparison to both control smokers and non-smokers (P < 0.001). However, smoking history did not influence eATP level in patients with COPD, as no significant difference between COPD patients subdivided according to their smoking status was observed when they were compared to each other (Fig. 1).

### GOLD classifications and eATP concentration

**Figure 2:**
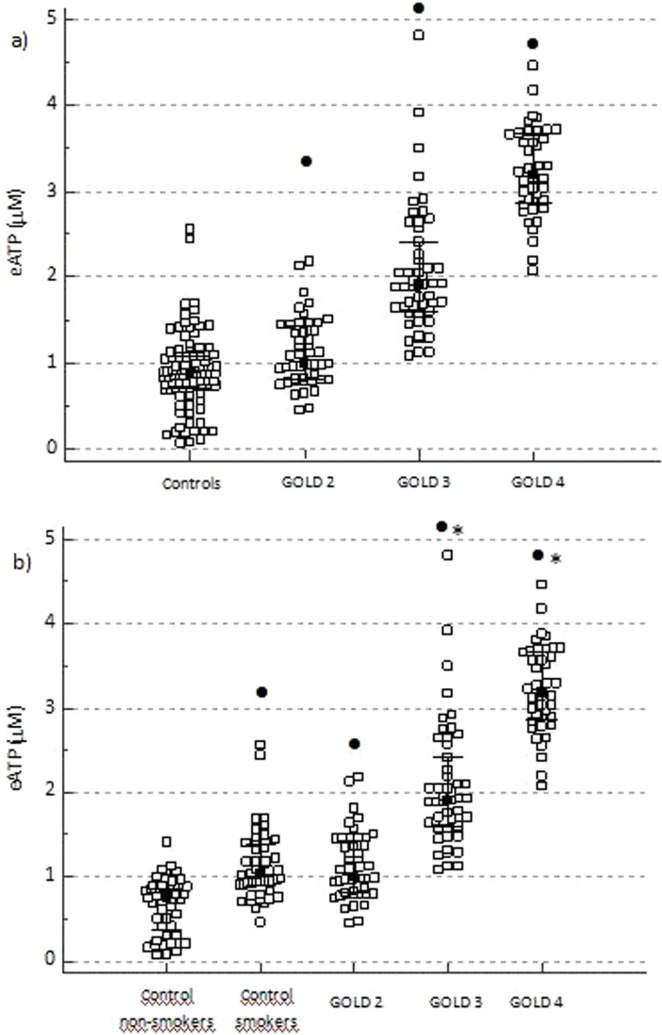
Influence of airflow limitation severity (GOLD 1–4 classification assessment) on eATP concentration. (**a**) eATP concentration was measured in EDTA plasma of control subjects and COPD patients subdivided by severity of airflow limitation into GOLD 2, GOLD 3 and GOLD 4 groups. (**b**) COPD patients were subdivided into GOLD 2–4 stages and compared to healthy individuals based on their smoking status (healthy non-smokers and healthy smokers). Data are shown as median with interquartile range for all the groups. Kruskal-Wallis test showed there was a significant difference between the groups (P < 0.001 for both **a,b**), and post-hoc analysis was performed. In COPD patients, a statistically significant increase in GOLD 3 in comparison to GOLD 2, and in GOLD 4 in comparison to GOLD 2 and GOLD 3 stages was also found. ^•^Statistically significant difference in comparison to total controls (**a**) or control non-smokers (**b**); *statistically significant difference in comparison to control smokers (**b**).

Next, we wanted to explore influence of airflow limitation severity as well as symptoms and exacerbations on eATP levels in peripheral blood. When patients were classified according to the airflow limitation severity characterized by FEV_1_ (GOLD 2, 3 and 4 stages; no patients fulfilled conditions for GOLD 1 group in our study), they all had significantly elevated plasma levels of eATP compared to controls. More importantly, concentration of eATP was significantly increasing with the severity of airflow limitation (Fig. 2a). When control subjects were subdivided due to their smoking history, patients with GOLD 3 and GOLD 4 stages had increased eATP concentration in comparison to both control non-smokers and control smokers (P < 0.05); however, difference was not observed between healthy smokers and patients in GOLD 2 stage (Fig. 2b).

**Figure 3:**
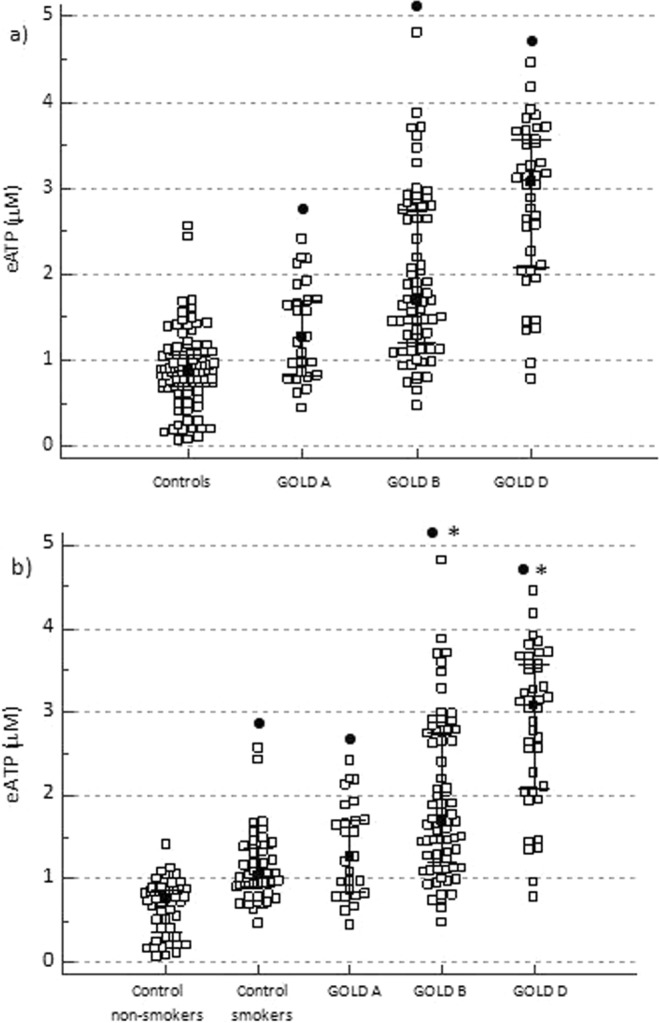
Influence of symptoms and history of exacerbations (GOLD A–D classification assessment) on eATP concentration. (**a**) eATP concentration was determined in EDTA plasma of control individuals and patients with COPD subdivided into GOLD A, GOLD B and GOLD D groups. (**b**) COPD patients were subdivided into GOLD A - D groups and compared to healthy subjects according to their smoking status (healthy non-smokers and healthy smokers). Data are shown as median with interquartile range for all the groups. Kruskal-Wallis test showed there was a significant difference between the groups (P < 0.001 for both **a,b**), and post-hoc analysis was performed. In COPD patients, a statistically significant increase in GOLD B in comparison to GOLD A, and in GOLD D in comparison to GOLD A and GOLD B groups was also found. ^•^Statistically significant difference in comparison to total controls (**a**) or control non-smokers (**b**); *statistically significant difference in comparison to control smokers (**b**).

COPD patients, when subdivided according to the symptoms burden and exacerbations history (GOLD A, B and D groups; no patients fulfilled conditions for GOLD C group in our study), had increased eATP concentration in comparison to the controls. Moreover, those three GOLD groups were significantly different from each other, and eATP was increasing with the severity of the symptoms (P < 0.05) (Fig. 3a). Also, eATP was increased in GOLD B and GOLD D patients’ groups when compared to both control non-smokers and control smokers (P < 0.05), while the levels of eATP did not differ between patients in GOLD A group and healthy smokers (Fig. 3b).

### Diagnostic value of eATP and correlation analysis

In order to evaluate eATP diagnostic performances, univariate logistic regression analysis was performed and OR of even 12.98 (95% CI = 6.10–27.62, P < 0.001) was obtained for eATP in EDTA plasma. In addition, 79% of cases were correctly classified by eATP measurement.

We also performed nonparametric measurement of rank correlation in COPD patients, and Spearman’s rank correlation coefficient (rho) and P-values were obtained (Table 2).

**Table 2:** Association between eATP and multicomponent indices as well as lung function parameters in COPD patients.

parameter	Spearman’s correlation coefficient, rho	P-value
ADO*	0.611	<0.001
BODCAT^▫^	0.785	<0.001
BODEx^•^	0.808	<0.001
CODEx^▪^	0.819	<0.001
DOSE°	0.765	<0.001
DLCO^◊^	−0.611	<0.001
FEV_1_^∆^ (L)	−0.764	<0.001
FEV_1_ (%)	−0.826	<0.001
FEV_1_/FVC^§^	−0.661	<0.001

Data were analysed by Spearman Rank Correlation. Results are described with Spearman’s correlation coefficient (rho) and P-value.

*ADO – age, dyspnoea, airflow obstruction; ^▫^BODCAT – BMI, airflow obstruction, dyspnoea, score from CAT; ^•^BODEx – BMI, airflow obstruction, dyspnoea, previous exacerbations; ^▪^CODEx – comorbidities (Charlson index), airflow obstruction, dyspnoea, previous exacerbations;

°DOSE – dyspnoea, airflow obstruction, smoking status, previous exacerbations; ^◊^DLCO – diffusion capacity for carbon monoxide; ^∆^FEV_1_ – forced expiratory volume in 1 second; ^§^FVC – forced vital capacity.

Previous exacerbations are defined as a number of exacerbations in the previous year.

Regarding basic spirometry parameters, eATP showed a very good to excellent negative correlation with FEV_1_ when expressed in litres and in percentage (%) of predicted values, and a moderate to good negative correlation with FEV_1_/FVC. DLCO, used for the assessment of the diffusion properties of the alveolar capillary membrane, negatively correlated moderately to well with concentration of eATP. When multicomponent indices ADO, BODCAT, BODEx, CODEx and DOSE that reflect patients’ condition (airflow limitation, dyspnoea, exacerbations, BMI, smoking, comorbidities and/or age) were correlated with eATP, they also showed a very good to excellent positive association, except ADO that demonstrated a moderate to good positive correlation with eATP concentration (Table 2).

### mRNA expression of eATP receptors

**Figure 4:**
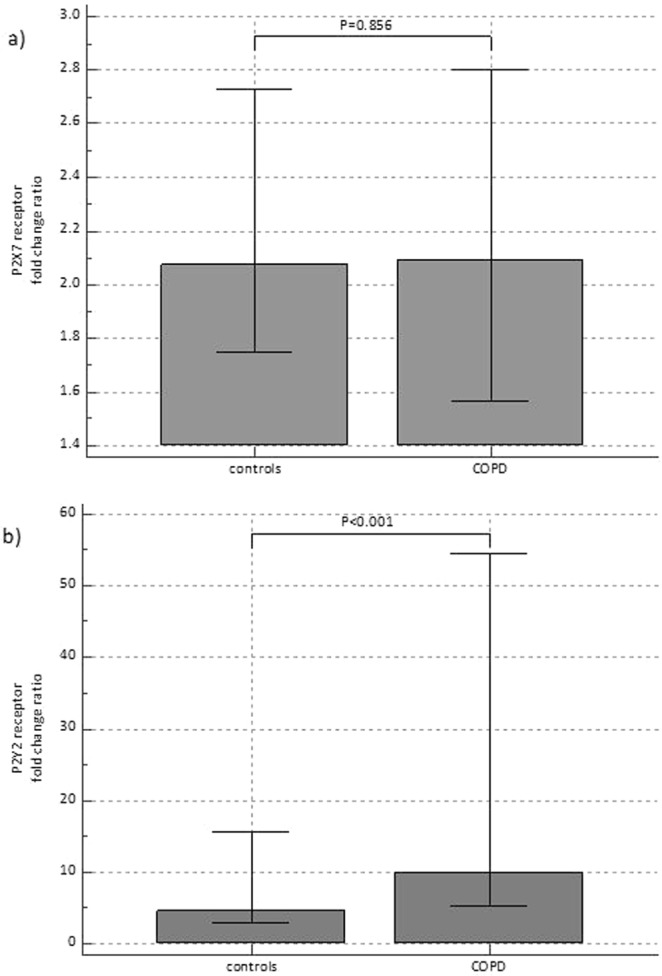
mRNA expression of eATP receptors P2X7R (**a**) and P2Y2R (**b**) in healthy subjects and patients with COPD. Results of gene expression are shown as fold change ratio with its median and interquartile range. Mann-Whitney test was used.

Finally, we explored mRNA expression of the two eATP receptors in the blood (buffy coat containing leukocytes and platelets) of participants from the study. The expression levels of P2X7R were similar in control and COPD patients (P = 0.856) (Fig. 4a), while P2Y2R showed twice as high expression in COPD patients in comparison to healthy individuals (P < 0.001) (Fig. 4b). The levels of P2Y2R mRNA expression did not differ between COPD non-smokers and healthy smokers (data not shown).

## Discussion

In this study, concentration of eATP was measured in the blood of COPD patients for the first time, and it was significantly elevated when compared to age- and sex-matched control subjects. Interestingly, eATP concentration differed between healthy non-smokers and smokers, and in COPD patients was associated with both airflow limitation severity (GOLD 1–4 stages) as well as with symptoms burden and history of exacerbations (assessed by GOLD A–D classification).

Different studies demonstrated an increase in eATP in media of cultured human bronchial epithelial cells^14^, BAL fluid of mice^12,33^, BAL fluid of COPD patients^9^ and EBC of COPD patients^7^, but no one measured eATP concentration in COPD patients’ blood samples – either plasma or serum. Serum is not an appropriate sample for quantification of eATP due to a potential interference of intracellular ATP from erythrocytes and platelets. Moreover, blood cell-derived ectonucleotidases in serum can affect eATP concentration^9^. Therefore, EDTA plasma was chosen as a representative sample of peripheral processes in COPD patients.

ATP could be released from different cells due to the cell injury or cell death. However, it can also be secreted through pannexin and connexin channels from intact activated cells (neutrophils, macrophages, platelets, epithelial and endothelial cells) as a result of nonlytic processes^2,14,15^. eATP acts as a damage-associated molecular pattern (DAMP)^34,35^, and we suggest that the levels of eATP determined in the blood of COPD patients might reflect multicomponent pathophysiological background of COPD.

Cigarette smoking is the most common underlying mechanism of COPD development, and COPD patients are mostly current or former smokers, which was confirmed by our study. Previously, an increase in eATP was observed in BAL of a mouse model of smoke-induced acute lung inflammation and emphysema^12,33^ as well as in BAL of COPD patients, even after smoking cessation^9,13^. In this study, control smokers had higher eATP concentrations in plasma from control non-smokers, although the levels were lower than in COPD patients, which might suggest some initial tissue changes (*e.g*. cellular destruction) and/or amplification of inflammatory responses in so-called healthy smokers. However, eATP concentrations in COPD patients were not associated with smoking history. Therefore, factors other than smoking seem to be primarily responsible for inducing eATP release that could aggravate ongoing inflammation in patients with COPD, making in such way a vicious circle.

It was reported that severity of airflow limitation and hypoxia are often associated with COPD exacerbations and could lead to ATP release from cells of the respiratory system^1^. In this study, eATP concentration in EDTA plasma was increasing with the disease severity based on airflow limitation, and the highest concentration was observed in GOLD 4 stage. Interestingly, there was no difference between control smokers and patients in GOLD 2 stage, while eATP in patients in GOLD 3 and GOLD 4 stages was increased in comparison to both control groups (smokers and non-smokers). Similar results were observed when symptoms and exacerbation history were assessed (GOLD A–D groups of patients), showing no difference between eATP levels in healthy smokers and patients in GOLD A group. This could become yet another reason for pro-active non-smoking campaign, as seems plausible that less severe COPD patients have similar pathophysiologic background with smokers who did not develop COPD. Naturally, pathogenesis gets more complex with the severity of the disease. Moreover, COPD patients from our study had lower value of pO_2_ (mmHg) = 70 (68–71) and normal value of sO_2_ (%) = 96 (95–98). Although we could not say with certainty if the COPD patients from our research were or were not hypoxic, eATP showed an association with oxygenation status. Indeed, eATP showed poor negative correlations with both parameters used for the assessment of oxygenation status (rho = −0.486, p < 0.001 and rho = −0.471, p < 0.001 for pO_2_ and sO_2_, respectively). Therefore, increased levels of eATP in plasma of COPD patients could be, at least partly, due to their poor oxygenation status.

Increased amount of eATP in the lungs of COPD patients leads to the activation of purinergic receptors that are widely expressed across all cells in the lungs^3^. It was shown that when activated by eATP, P2Y2Rs lead to further ATP release by pannexin dependant mechanism, which then activates P2X7Rs. Although it was only investigated in viral, bacterial and protozoa infections, suggested cooperation between purinergic receptors driven by ATP could be observed in other conditions when immune response is needed due to inflammatory processes^36,37^. Careta *et al*. demonstrated that expression of P2X7R and P2Y2R were down-regulated in the lungs of non-obstructed smokers and COPD patients, but their study included patients in an early stage of COPD and a very few of them. Moreover, their study population had both lung cancer and COPD, so there might had been a bias regarding the observations associated with COPD^13^. Increased P2X7R expression in the lungs and BAL cells after LPS stimulation was reported^38^. In addition, P2X7R was up-regulated in BAL macrophages and blood neutrophils from patients with COPD^9^. On the other hand, P2Y2R showed an important role as a mediator in the recruitment of macrophages and neutrophils at the site of inflammation where damaged cells are being phagocytosed by macrophages and neutrophils. P2Y2Rs could induce hyperinflammation and tissue damage by promoting a chronic state of the disease by increasing activity and migration capacity of neutrophils^39^. Moreover, eATP acts like an autocrine messenger through activation of P2Y2Rs and amplifies chemotaxis signals^15^. The P2X7R is ATP-selective receptor, but it is considered to have a low affinity for ATP^40^. If the translocation of P2X7R to the cell membrane is regulated by eATP concentration at the transcriptional and/or post-transcriptional levels, it is possible that observed concentrations of eATP in patients with COPD were not high enough to activate P2X7Rs, since in our study mRNA expression of this receptor was not different in comparison to healthy subjects. On the other hand, mRNA expression level of P2Y2R was twice as high in patients with COPD in comparison to control individuals. This could reflect on higher activity of neutrophils, and we detected a significantly increased number of neutrophils in COPD patients (data not shown). What is also interesting is that the levels of P2Y2R mRNA expression did not differ between COPD non-smokers and healthy smokers, which confirms once again significant disturbances within healthy smokers’ organisms.

In our study, eATP with an OR of 12.98 showed a great predicting power, and correctly classified 79% of cases. In addition, eATP demonstrated a very good to excellent positive correlation with multicomponent indices BODCAT, BODEx, CODEx and DOSE that indirectly reflects an association with most of the major COPD-related factors, such as airflow limitation, dyspnoea, exacerbations, BMI, smoking, comorbidities and/or age. On the other hand, we also showed a very good to excellent negative correlation with FEV_1_, meaning that the severity of the disease was followed by an increase in eATP concentration, and it might be that eATP-driven inflammation is a part of the disease progression. There are several methods for the assessment of pulmonary function. Besides spirometry, in the present study DLCO was determined in COPD patients. As expected, it was decreased due to emphysema present in COPD and was decreasing with more advanced severity stages of disease (data not shown). The loss of alveoli in emphysema is resulting in smaller surface area available for diffusion of respiratory gases, so carbon monoxide (CO) transfer from the alveolar gas to the haemoglobin of the erythrocytes in the pulmonary circulation is also diminished^41^. Based on the results from this study, it could be suggested that eATP concentration was a reflection of decreased lung function, since there was a negative correlation between eATP and DLCO as well as FEV_1_ in COPD patients.

Limitation of this study is a lack of participants in GOLD 1 stage and GOLD C group of COPD patients. It would be interesting to investigate eATP at the beginning of the disease development (GOLD 1 stage), but, unfortunately, this group of COPD patients rarely contacts physicians in our outpatient clinic due to very mild symptoms. In addition, GOLD C category of patients is also very rare, as patients that do not have many symptoms usually are not frequent exacerbators. Also, the sample size was small for group of COPD non-smokers. As we found that P2Y2R expression is similar between this subgroup of patients and healthy smokers, it would be interesting to confirm that the observed effect was not just a bias due to an inadequate number of patients. Therefore, a prospective study with more participants is suggested.

In conclusion, eATP concentration in EDTA plasma of COPD patients in stable phase showed great diagnostic performances and was associated to the disease progression described by airflow limitation severity as well as symptoms and exacerbation history. Moreover, it might be that smoking is a part of eATP-driven systemic inflammation, especially in healthy smokers, who had increased levels of eATP when compared to control non-smokers, their P2Y2R expression levels were similar to COPD non-smokers, and their eATP concentration was similar to COPD patients in GOLD 2 stage as well as in GOLD A group. Blood is an easily and non-invasively obtained sample, and plasma eATP could become a diagnostic and/or prognostic biomarker in COPD, as it seems to be associated with patients’ condition, quality of life and disease progression.

## Data Availability

All data generated and/or analysed during the current study are available from the corresponding author on reasonable request.
